# Prevalence of Burnout Syndrome and other psychiatric disorders among health professionals during the COVID-19 pandemic: A systematic review and meta-analysis protocol

**DOI:** 10.1371/journal.pone.0260410

**Published:** 2021-12-02

**Authors:** Kleyton Santos de Medeiros, Letícia Maniçoba Ferreira de Paiva, Luíza Thomé de Araújo Macêdo, Wederson Farias de Souza, Luís Antônio Soares da Silva, Ayane Cristine Alves Sarmento, Ana Paula Ferreira Costa, Cijara Leonice Freitas, Ana Katherine Gonçalves

**Affiliations:** 1 Health Sciences Postgraduate Program, Federal University of Rio Grande do Norte (UFRN), Natal, Rio Grande do Norte, Brazil; 2 Instituto de Ensino, Pesquisa e Inovação, Liga Contra o Câncer, Natal, Rio Grande do Norte, Brazil; 3 Department of Nursing, Centro Universitário do Rio Grande do Norte, Natal, Rio Grande do Norte, Brazil; 4 Department of obstetrics and Gynecology, Federal University of Rio Grande do Norte (UFRN), Natal, Rio Grande do Norte, Brazil; Xiamen University - Malaysia Campus: Xiamen University - Malaysia, MALAYSIA

## Abstract

**Introduction:**

Studies carried out during previous pandemics revealed an increase in the prevalence of Burnout Syndrome and other psychiatric disorders among health professionals. A high prevalence of psychiatric disorders is also observed in some health categories, during the COVID-19 pandemic.

**Objective:**

This systematic review/meta-analysis study aims to assess the prevalence of Burnout Syndrome and other psychiatric disorders (depression, anxiety, stress, and insomnia) among health care professionals and other support professionals during the COVID-19 pandemic.

**Inclusion criteria:**

Observational studies published from December 2019, without language restrictions in which the prevalence of Burnout Syndrome and other psychiatric disorders among health professionals during the COVID-19 pandemic will be assessed.

**Methods:**

PubMed, Web of Science, Embase, CINAHAL, PsycINFO, LILACS, SCOPUS, and The Cochrane Library will be searched for eligible studies. Two reviewers will independently screen and select studies, assess methodological quality, and extract data. A meta-analysis will be performed, if possible, and the Grading of Recommendations Assessment Development and Evaluation (GRADE).

**Ethics and disclosure:**

This study will use secondary data. Thus, there is no need for submission to the ethics committee. The results of this systematic review will be published in a journal after a peer-review process.

**Trial registration:**

**Systematic review registration number:** CRD42020212036.

## Introduction

The coronavirus disease (COVID-19) has been affecting every country in the world drastically [1–4]. According to the World Health Organization (WHO, 2020), more than 198,032,883 cases of COVID-19 were detected and around 4,220,504 deaths occurred worldwide [5]. In view of this context, high levels of anxiety, stress, depression, and Burnout Syndrome are already observed in the general population, inclusive health professionals and support professionals who care for patients with COVID-19.

Both health professionals directly involved in the diagnosis, treatment, and care of patients with COVID-19, as support professionals, that work together with these professionals and health institutions are at high risk of developing psychological distress and other mental health symptoms. This is because they work under extreme pressure, are exposed to high levels of stress, work prolonged shifts, have excessive workload, sometimes work without training, and often do not receive appropriate personal protective equipment. Moreover, they face unprecedented situations, such as allocating scarce resources to equally needy patients, providing assistance with restricted or inadequate resources, and a lack of specific drugs [6, 7]. These conditions can trigger feelings of loneliness and helplessness, or a series of emotional states, such as stress, irritability, physical and mental fatigue, and despair. Work overload and symptoms related to stress make health professionals especially vulnerable to psychological suffering, which puts them at increased risk for developing psychiatric disorders [8, 9].

Throughout history, it has been observed that during a health crisis, health teams tend to mobilize more actively, causing them to forget about the risk transmissibility of the infection. For example, during the severe acute respiratory syndrome (SARS) outbreak in 2003, 18% to 57% of health professionals presented serious emotional problems and psychiatric symptoms during and after the event [10]. In 2015, during Middle East respiratory syndrome, depression and stress were observed among health professionals. Frontline professionals were demonstrated to be at a higher risk of developing post-traumatic stress disorder (PTSD). There are also studies that show increased levels of stress, depression, anxiety, and PTSD among professionals even after some time had transpired since the end of the outbreak [11, 12].

Some studies performed in order to explore the psychiatric repercussions in health professionals during the COVID-19 pandemic presented significant results. Elbay et al. (2020) reports that according to data collected in China during the COVID-19 pandemic, health professionals had a high prevalence of depression, being reported by 50% of the professionals interviewed [13]. Zhang et al. (2020) also demonstrate that health workers, including doctors, showed a higher prevalence of insomnia, anxiety, depression, somatization, and obsessive-compulsive symptoms [14]. In another study conducted by Lai et al. (2020), a large number of participants presented symptoms of depression, anxiety, insomnia, and distress [15]. Finally, Song et al. (2020), found prevalence rates of depressive symptoms of 25.2% among 14,825 doctors and nurses in 31 provinces of mainland China [16].

However, we have not identified a comprehensive systematic review, about the psychiatric disorders, beyond the depression, such as Burnout Syndrome, anxiety, stress, and insomnia presented in health professionals during a pandemic. For this reason, this systematic review/meta-analysis study aims to assess the prevalence of Burnout Syndrome and other psychiatric disorders (depression, anxiety, stress, and insomnia) among health care professionals and other support professionals during the COVID-19 pandemic.

### Review questions

What is the prevalence of Burnout Syndrome and other psychiatric disorders, such as depression, anxiety, stress, and insomnia, among health care professionals and other support professionals during the COVID-19 pandemic worldwide?

## Materials and methods

### Protocol and registration

This protocol is registered with the International Prospective Register of Systematic Reviews (PROSPERO) under the CRD number CRD42020212036.

The proposed systematic review and meta-analysis conforms to the Meta-analysis of Observational Studies in Epidemiology [17] and Preferred Reporting Items for Systematic Reviews and Meta-Analyses (PRISMA-P) [18] guidelines.

### Inclusion criteria

This systematic review protocol will include the following studies:

### Participants

- Studies on health professionals (doctors, nurses, physiotherapists, pharmacists, nutritionists, paramedics, technicians, and allied health occupations) and development of Burnout Syndrome and other psychiatric disorders (depression, anxiety, stress, and insomnia) during the COVID-19 pandemic;
- Studies published from December 2019 until July 2022.

### Exposure

- Health professionals and allied health occupations involved in combating COVID-19.

### Outcome

- The outcome of interest is the prevalence of Burnout Syndrome and other psychiatric disorders (depression, anxiety, stress, and insomnia) among health professionals during the COVID-19 pandemic, which will be evaluated by primary studies from the scales: Patient Health Questionnaire-9 (PHQ-9) [19, 20]; Depression Anxiety Stress Scales (DASS-21) [21, 22], Self-Rating Depression Scale (SDS) [23, 24], Beck Depression Inventory (BDI) [25, 26], Insomnia Severity Index (ISI) [14], Revised Symptom Checklist (SCL-90-R) [14] and Patient Health Questionnaire-4 (PHQ-4) [14], Generalized anxiety disorder 2-item (GAD-2) [27], Stanford Sleepiness Scale [28] and other scales that can be found in the articles to be selected.

### Types of studies

Only specific human observational study designs will be included such as: longitudinal cohort studies (prospective and retrospective), cross-sectional studies, and case-control studies. Case series and case reports will be excluded due to their low level of scientific evidence. Randomized controlled trials and quasi-experiments will also be excluded because this review does not examine the role of any intervention related to depression. There will be no language restrictions when selecting studies.

### Exclusion criteria

1. Case reports, case studies, letters to the editor, fact sheets, conference abstracts and review articles.
2. Studies with children and adolescents <18 years.
3. Studies with the general population, except healthcare professionals.

### Information sources

A search will be conducted in the following databases: PubMed, Web of Science, Embase, Cumulative Index to Nursing and Allied Health Literature (CINAHL), PsycINFO, Latin American and Caribbean Literature in Health Sciences (LILACS), SCOPUS, and The Cochrane Library, will be searched for articles dated between December 2019 and July 2022. The reference lists will be screened. The search strategy will be to use the medical subject headings (MeSH) and terms that have been included in Table 1. The literature screening will be performed by four reviewers.

**Table 1:** Medline search strategy.

Search items	
1	Health Professions
2	Health Occupation
3	Nurses
4	Physicians
5	Doctor
6	Physiotherapist
7	Physiotherapist
8	Nutritionists
9	Pharmacists
10	Paramedics
11	Technicians
12	Allied Health Personnel
13	Allied Health Occupations
14	Mental disorder
15	Depressive Disorder
16	Syndrome Depressive
17	Depression
18	Anxiety
19	Acute Stress Disorders
20	Burnout Syndrome
21	Insomnia disorder
22	Insomnia
23	COVID-19
24	SARS-Cov-2
25	Observational Study
26	Cross-sectional Studies
27	Cohort Studies
28	Case-control Studies

### Search

The terms of the MeSH will be: ((Health Professions OR Health Occupation OR Nurses OR Physicians OR Doctor OR Physiotherapist OR Nutritionists OR pharmacists OR paramedics OR Technicians OR Allied Health Personnel OR Allied Health Occupations) AND (Mental disorder OR Depressive Disorder OR Syndrome Depressive OR Depression OR anxiety OR Acute Stress Disorders OR Burnout Syndrome OR insomnia disorder OR insomnia) AND (COVID-19 OR SARS-Cov-2)) (Table 1).

### Study selection

**Fig 1:**
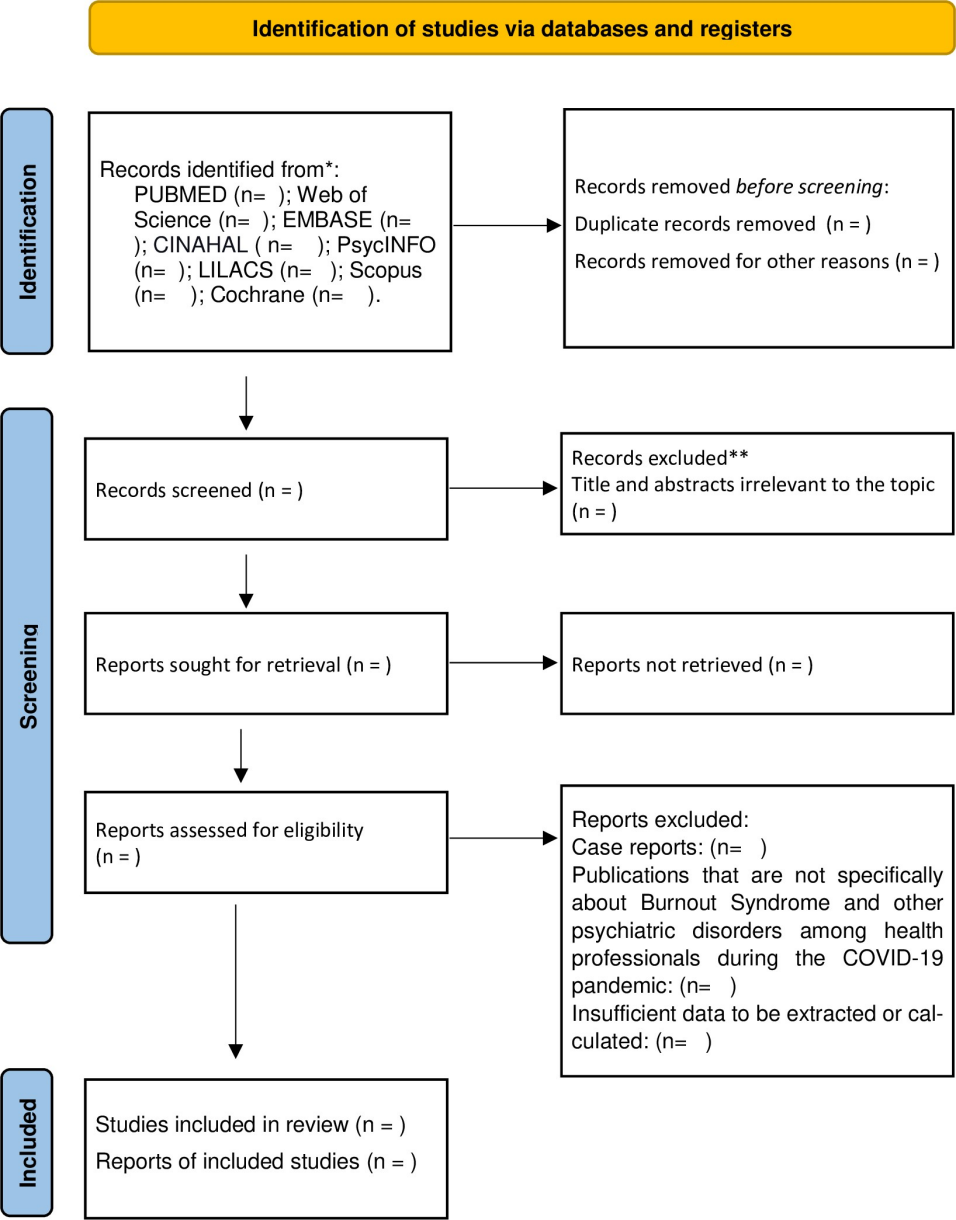
Flow diagram of the search for eligible studies on the prevalence of Burnout Syndrome and depression, anxiety, stress, and insomnia among health professionals during the COVID-19 pandemic: CENTRAL, Cochrane central register of controlled trials.

Five authors, KSM, LMFP, WFS, LTAM and LASS will independently select the articles, using titles and abstracts. Duplicate studies will be excluded. The same authors will review the text to determine if the studies meet the inclusion criteria. A sixth reviewer, AKG, will resolve the discrepancies. The selection of studies will be summarized in a PRISMA flow diagram (Fig 1).

### Data collection process

A standardized data extraction form will be developed and tested. Data from each included study will be extracted independently by three reviewers (ACAS, APFC and CLF), and any subsequent discrepancies will be resolved through discussion with a fourth reviewer (AKG). The data extracted will include information on authors, year of publication, study location (country and continent), type of study, main objectives, population, depression, anxiety and stress assessment (PHQ-9, DASS-21, SDS, BDI and others scales) [19–26], risk factors, protective factors, use of medications, biological variables, treatment, and patient outcomes. Furthermore, participant characteristics (e.g., mean age, gender), and results for the prevalence will be collected.

The study authors will be contacted in case of missing data and/or to resolve any uncertainties. In addition, any additional information will be recorded. All data entries will be checked twice. If we find a set of articles with similar characteristics based on the information in the data extraction table, we will perform a meta-analysis using a random-effects model. If there is data that is not clear in some articles, the corresponding author will be contacted for possible clarification.

### Assessment of methodological quality

The methodological quality of each included study will be assessed by two reviewers (KSM and APFC) independently. They will do so using a widely-recognized standardized critical appraisal instrument from the Joanna Briggs Institute for the following study types: cross-sectional [29]. Study authors will be contacted in the event of insufficient details to confidently assess the methodological quality.

The risks of bias of observational (cohort and case-control) studies will be assessed by two reviewers (KSM and ACAS) independently using Newcastle–Ottawa Scale tool. The quality of each cohort study was judged on three broad categories—namely, selection of the study population, comparability of groups and ascertainment of either the exposure or outcome of interest [30].

The tool evaluates biases from confounding factors, selection of participants into the studies, missing data, and measurement of outcomes. Any unresolved disagreements will be resolved through discussion and/or consensus with a third reviewer (AKG). Study authors will be contacted in the event of insufficient details to confidently assess the risk of bias.

### Assessing certainty in the findings

The quality of evidence for all outcomes will be assessed using the Grading of Recommendations Assessment, Development, and Evaluation (GRADE) approach [31]. The quality of the evidence will be assessed based on the risk of bias, indirectness, inconsistency, imprecision, and publication bias.

### Synthesis of results

We will use the Review Manager statistical software V.5.2.3 to analyze the data. Some degree of heterogeneity is expected across the studies; therefore, a random-effects model for meta-analysis will be applied. Effect sizes will be expressed as risk ratios, odds ratios, or prevalence ratios (for dichotomous data) and weighted (or standardized) mean differences (for continuous data) and their 95% confidence intervals (CI) will be calculated. Cohort estimates will be presented as risk ratios or prevalence ratios with 95% CI, and case-control estimates will be presented as odds ratios with 95% CI. The degree of statistical heterogeneity will be assessed using standard I^2^ squared statistics. Where statistical pooling is not possible and/or there is substantial heterogeneity, we will provide a narrative synthesis of the study findings. Sensitivity analyses will be performed to explore the impact of the quality of the included studies. Publication bias will be assessed if more than 10 studies were included using a funnel plot. To assess funnel plot asymmetry, Egger’s test (for continuous outcomes) will be performed.

## Discussion

Health professionals that work at the frontline of combating COVID-19, and the support professionals that work together with these professionals and health institutions are at high risk of developing psychological distress and other mental health symptoms [30, 31]. Several factors can cause psychological distress in these professionals, once during situations of a pandemic, the psychological needs of these professionals are neglected [32].

According to Zhang et al. (2020), during the COVID-19 pandemic, the professional’s health had a higher prevalence of insomnia, anxiety, depression, somatization, and obsessive-compulsive symptoms on the account. Dealing with psychological distress and the risk of allostatic overload [14]. Lai et al. (2020) also showed that a considerable proportion of participants related symptoms of depression, anxiety, insomnia, and distress to the contacted individuals in their study. Nurses, women, frontline health care workers, and those working in Wuhan, China, reported more severe degrees of mental health distress [15]. In addition, Song et al. (2020) found that medical and nursing staff workers in Hubei province were associated with a higher risk of depressive symptoms, while those working in the Hubei province but residing in another province had a lower risk of depressive symptoms and PTSD. All the studies pointed out the importance of implementing psychological interventions to promote mental health among these professionals [16].

One cross-sectional study conducted in India among healthcare workers directly involved in screening, diagnosing, and treating COVID-19 patients and suspects, the prevalence of health professionals with high-level stress was 3.7%, while the prevalence rates of professionals with depressive symptoms requiring treatment and anxiety symptoms requiring further evaluation were 11.4% and 17.7%, respectively. Women had approximately twice the increased odds of developing high-level moderator stress, depressive symptoms requiring treatment, and anxiety symptoms requiring further evaluation [33]. In Brazil, a survey conducted using social media and administrative emails to Brazilian active healthcare professionals during the COVID-19 outbreak showed that new insomnia symptoms or previous insomnia worsening occurred in 41.4% of the professionals. Prevalent anxiety and burnout during the pandemic were observed in 44.2% and 21% of participants, respectively. Multivariate analyses showed that females, weight change, prevalent anxiety, new-onset burnout, and family income reduction >30% were independently associated with new-onset or worsening of previous insomnia [34].

The countries with the highest number of COVID-19 cases and deaths were the USA, Brazil, and India, where working conditions differ from those in China. Additionally, socio-cultural differences and socioeconomic disparities may be related to the development of depression among health professionals [35]. Another factor to consider is geographic location, which was also a risk factor in Italy’s study that compared stress and anxiety between healthcare workers and the general population [36]. A study capable of identifying countries with a higher number of health professionals with depression will promote adoption of strategies to prevent and treat the disease, thereby allowing these professionals to not have to leave their work activities.

The previous systematic reviews presented some weaknesses that justify the realization of new studies. For example, Pappa et al (2020) [37] sought studies until April 2020, after which there was a peak in the pandemic in several other countries, which may have increased the prevalence of depression. Further, the latter did not focus solely on assessing the prevalence of depression and mainly included studies with professionals from China, with no data from other countries with a higher number of cases. Already, Li et al (2021) [38], did not evaluate results for insomnia and burnout. Thus, a systematic review that evaluates the real prevalence of psychiatric disorders, such as Burnout Syndrome, depression, anxiety, stress, and insomnia, among health professionals and support professionals is essential for preventive and therapeutic measures focused on specific groups based.

Among the study’s limitations, the possibility of high heterogeneity between studies stands out, the different scales and cutoff points for the assessment of outcomes. Furthermore, different population samples in different regions of the planet in which working conditions, access to personal protective equipment, and the professional’s technical capacity may vary, resulting in a greater or lesser prevalence of outcomes.

## Supporting information

S1 Checklist Preferred Reporting Items for Systematic review and Meta-Analysis Protocols (PRISMA-P checklist). (DOCX)
